# Moral courage level of nurses: a systematic review and meta-analysis

**DOI:** 10.1186/s12912-024-02082-w

**Published:** 2024-08-02

**Authors:** Hang Li, JuLan Guo, ZhiRong Ren, Dingxi Bai, Jing Yang, Wei Wang, Han Fu, Qing Yang, Chaoming Hou, Jing Gao

**Affiliations:** 1 Chengdu University of Traditional Chinese Medicine, Chengdu, Sichuan China; 2 Hospital of Chengdu University of Traditional Chinese Medicine, Chengdu, Sichuan China; 3 The Affiliated Fifth People’s Hospital of Chengdu University of Traditional Chinese Medicine, Chengdu, Sichuan China

**Keywords:** Moral courage, Moral distress, Nurses, Meta-analysis

## Abstract

**Background:**

Moral distress occurs in daily nursing work and plagues nurses. Improving the level of moral courage is one of the main strategies to reduce moral distress, and low levels of moral courage may lead to nurse burnout, increased turnover, and reduced quality of care.

**Methods:**

Nine electronic databases in Chinese and English were searched for the level of moral courage among nurses, including PubMed, Web of Science, EMBASE, CINAHL, CNKI, Wan fang, Wei pu, CBM and Cochrane Library, for the period from the date of database creation to April 5, 2023. The Agency for Healthcare Research and Quality (AHRQ) was used to assess the methodological quality of the included studies, followed the Preferred Reporting Items for Systematic Reviews and Meta-Analyses (PRISMA) guidelines and the Meta-analysis and Systematic Reviews of Observational Studies guidelines, and data from the included studies were meta-analyzed in STATA version 15 using a fixed-effects model.

**Results:**

Seventeen cross-sectional studies of moderate or high quality met the eligibility criteria and involved 7718 nurses, and the Nurses’ Moral Courage Scale (NMCS) was used to measure the self-assessed moral courage level of nurses. Eleven of these studies reported total scores for nurses’ moral courage, and the meta-analysis results showed a pooled mean score of 78.94 (95% CI: 72.17, 85.72); Fourteen studies reported mean entry scores for nurses’ moral courage, and the meta-analysis results showed a pooled mean score of 3.93 (95% CI: 3.64, 4.23).

**Conclusion:**

The results of the meta-analysis showed that nurses’ moral courage levels were in the medium to high range, among the nurses who seemed to be male, non-nursing managers, high school education, had not experienced ethical issues, and considering resignation had lower levels of moral courage. The results of the meta-analysis may provide some reference for nursing managers and even hospital administrators to develop strategies to optimize nursing quality.

**Supplementary Information:**

The online version contains supplementary material available at 10.1186/s12912-024-02082-w.

## Introduction

Nursing is embedded in ethical and moral concerns [1, 2], The survey showed that 67.7% of nurses experience moral distress [3], and that distress is more frequent and more serious for nurses than for other healthcare workers because they have more contact time with patients, so the frequency of moral distress is relatively high [4], especially during disease pandemics, which creat more ethical issues and distress for nurses, increasing their moral suffering [5, 6]. Research suggests that moral distress negatively affects nurses, for example, when nurses are in a chronic moral distress, it decreases their job satisfaction and increases turnover rates [7]; it can also lead to empathic fatigue [8], burnout [9], and an increased rate of medication errors among nurses [10].Therefore healthcare organizations must recognize the negative effects of moral distress on nurses and take proactive measures in order to mitigate its impact on both individuals and patient outcomes.

Improving the level of moral courage is one of the main strategies to reduce the frequency of moral distress [11]. Moral courage is the courage to act in accordance with moral principles in the face of moral conflict, even though one may experience negative consequences [12], and in the field of nursing, moral courage defined as the nurse’s ability to adhere to professional ethical guidelines and to act in strict compliance with those guidelines, even if there is a foreseeable or real negative impact on yourself as a result [13]. Research [14]shows that nurses with higher level of moral courage experience lower frequencies of moral distress. High level of moral courage enables nurses to effectively respond to challenging situations and uphold their professional values. Additionally, high moral courage enables them to openly oppose unethical practices, protect patients’ rights and make the right decisions. Low level of moral courage may lead to nurses being unable to adhere to ethical principles, leading to an increase in the frequency of moral distress, thereby reducing the quality of care, and ultimately leading to unethical behavior [15]. As the backbone of the healthcare system, nurses require a supportive environment to meet their needs [14].

Encouragingly, scholars are increasingly paying attention to nurses’ current level of moral courage. Therefore, the number of studies on this topic is gradually increasing. However, it is worth noting that there is a wide range of opinions regarding the level of moral courage exhibited by nurses. Tang et al. [16] surveyed 331 psychiatric nurses in a hospital in Henan Province and the study showed that the moral level of nurses was at a higher level. Other studies have reached different conclusions, for example, Gan et al. [17] surveyed 368 junior nurses in a hospital in Harbin and showed that nurses’ moral courage was at a moderate to low level, and Nora Hauhio et al. [18] surveyed 482 registered nurses in a hospital in Finland and showed that nurses’ moral courage was at a moderate to high level, which may be related to the survey area, sample size, and the nurse’s work environment, work experience, and education level [13, 19, 20]. Although different studies have drawn different opinions and conclusions, one thing is still certain - nurses are an indispensable part of maintaining ethical standards in the medical field. Their role cannot be overemphasized, as they are often at the forefront of patient care and promotion. Therefore, we must study the current situation of nurses’ moral courage so that we can identify areas for improvement to enhance their level of moral courage. This not only helps to reduce the ethical distress faced by nurses, but also helps to improve the overall quality of care [21].

To date, our search of major databases revealed that there are no meta-analyses of nurses’ levels of moral courage, indicating a lack of evidence-based evidence in this area. Therefore, the purpose of this review is to understand the level of moral courage of nurses by pooling studies which using NMCS.

## Methods

### Design and registration

The Systematic review and Meta-analysis followed the Preferred Reporting Items for Systematic Reviews and Meta-Analyses (PRISMA) guidelines [22] and the Meta-analysis and Systematic Reviews of Observational Studies guidelines [23], it can enhance the clarity and organization of reports, so the systematic reviews and meta-analysis reports will not miss important information, thus providing high-quality evidence for evidence-based decisions. This systematic review and meta-analysis have been registered on PROSPERO website (Registration number: CRD42023414565).

### Search strategy

The studies were searched in nine electronic databases in English and Chinese (PubMed, Web of Science, EMBASE, CINAHL, CNKI, Wan fang, Weipu, CBM and Cochrane Library), for the period from the date of database creation to April 5, 2023. A combination of Mesh terms and free terms was used for the literature search. The Mesh terms included “Nurses”, “Nurse”, “Nursing Personnel”, “Registered Nurses”, and “Moral Courage”. To ensure the comprehensiveness of the literature search, references cited in the literature were manually searched to find the research that may be included in the literature. We will also seek the help of an experienced librarian to refine the search strategy for each database. For full text not available or only abstracts or unpublished documents, we will email the corresponding author or first author for help. (Supplementary Table 1)

### Eligibility criteria

Inclusion criteria:

1. The research subjects included in the study are nurses.
2. The Nurses’ Moral Courage Scale was developed by Numminen et al. [24] in 2018 to assess the level of moral courage.
3. It is a quantitative study that can extract the mean ± standard deviation of the total score of the scale or the mean ± standard deviation of the mean score of each item.
4. Observational studies (cross-sectional, case-control, cohort studies).

Exclusion criteria:

1. Unable to extract mean ± standard deviation of scale scores.
2. Secondary research (Meta-analysis, Systematic evaluation, Review, etc.).
3. Full text was not available.

### Quality assessment

Since all studies included in this review are cross-sectional the Agency for Healthcare Research and Quality (AHRQ) was used to assess methodological quality [25], which is currently an excellent tool for assessing the quality of cross-sectional studies [26]. It is also one of the widely accepted tools for assessing the quality of cross-sectional studies, and the AHRQ is available at http://www.ncbi.nlm.nih.gov/books/NBK35156/ [27]. The AHRQ has 11 items and assigns a score of 1 when assessing individual items for “yes” and 0 points otherwise. The total score is 0 to 3 for low quality, 4 to 7 for moderate quality, and 8 to 11 for high quality. This study quality was assessed by the LH reviewer and then checked by the reviewer BDX, and any discrepancies were resolved through discussion. (Supplementary Table 2)

### Data extraction

Two researchers (LH and FH) independently selected the literature in EndNote X9, extracted the data, and cross-checked according to search strategies and inclusion criteria. In case of any disagreement, a third researcher (BDX) was consulted for resolution. The main data were extracted in Microsoft Office Excel, including: Study, Country, Study design, Total sample, Number of Male, Number of Female, Age, Moral courage score, Average score of entries. (Table 1)

### Data synthesis

All included studies used a consistent measurement instrument, so meta-analysis was used to synthesize the quantitative data. Mean scores and standard deviations of NMCS scale scores were pooled across studies using Stata15 software, and the pooled mean scores were expressed as weighted effect sizes and 95% confidence intervals (CI). Between-study heterogeneity was assessed using the Cochran *Q* chi-square test and the *I*^*2*^ statistic, with *I*^*2*^ values of 25%, 50%, and 75% for low, moderate, and high heterogeneity, respectively. When *I*^*2*^ > 50% and *p* < 0.05, moderate or high heterogeneity was indicated and a random effects model was used for analysis; otherwise, a fixed effects model was used [28]. In addition, pre-defined subgroup analyses were used to explore the effects of gender, whether or not they were nurse leaders, education level, on the level of nurse ethics, and whether or not they were experiencing ethical problems or related knowledge. Egger’s test was used to identify publication bias, with *P* > 0.05 indicating a low likelihood of publication bias [29]. If publication bias exists, correction is made by haircutting.

## Results

### Study screening & selection process

**Fig. 1:**
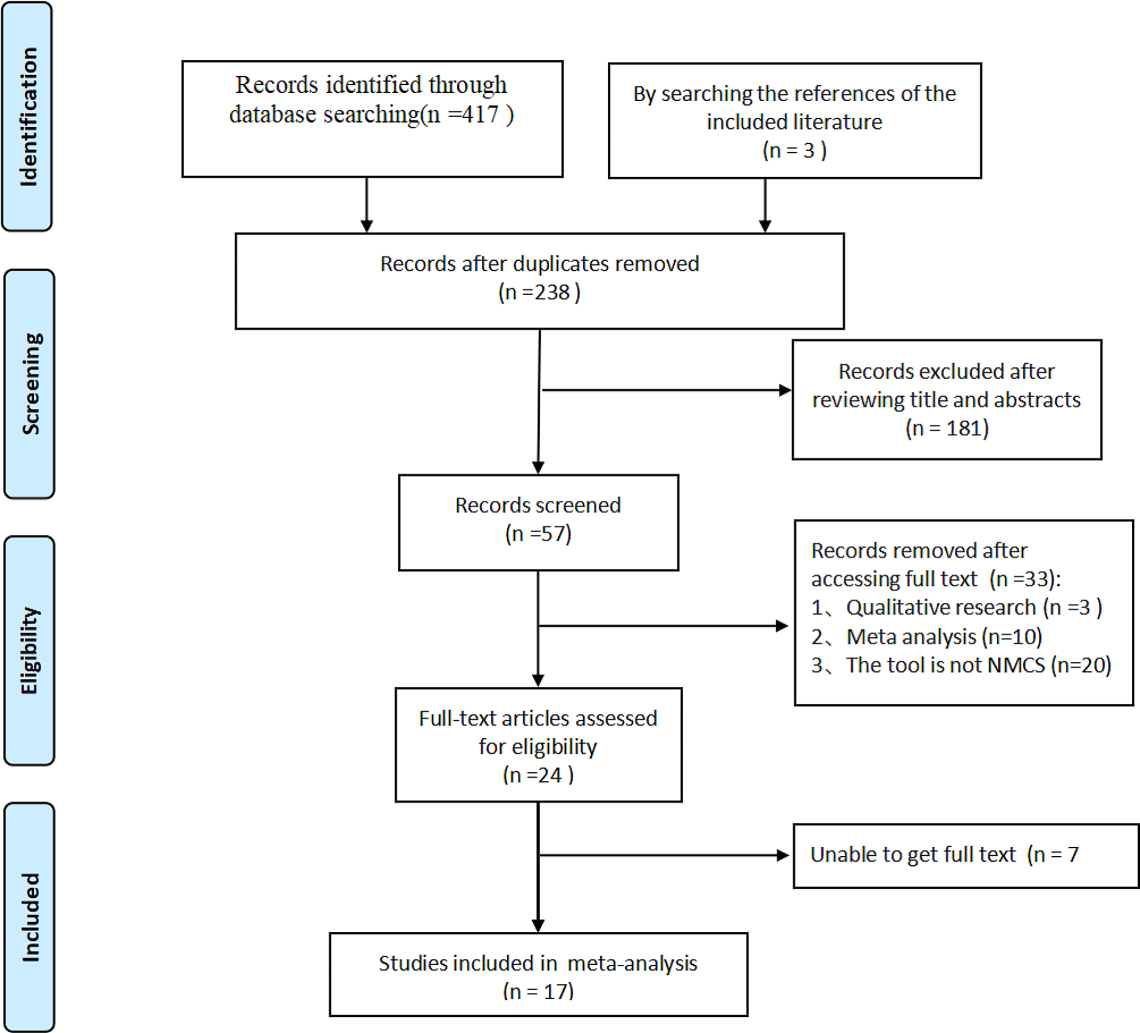
The literature screening flow chart

417 literature were obtained through database search, 3 literature obtained by tracing the included references, obtained a total of 420 literature. According to the inclusion and exclusion criteria, 181 obviously irrelevant literature were excluded from the initial screening; after reading the full text and re-screening, 40 literature with inconsistent study subjects, study content, study design, outcome indicators, non-English and Chinese, non-accessible full text were excluded, and 17 [16–18, 30–43] citations were finally included. (Fig. 1)

### Study description

A total of seventeen studies involving 7718 nurses were included in this review, eleven of these studies reported total scores for nurses’ moral courage, fourteen studies reported mean entry scores for nurses’ moral courage, we separately merged the mean standard deviation of the total score of moral courage level and the average score of each item to better review the current status of nurses’ moral courage level. The included studies were all published between 2020 and 2023; a few studies (n = 5) were conducted in European countries (Finland, Turkey, Belgium), while the majority (n = 11) were conducted in China, and all studies were cross-sectional. The included studies all used the Nurse Moral Courage Scale developed by Numminen et al [24]. The scale consists of 21 items in 4 dimensions, namely moral integrity (7 items), commitment to good care (5 items), compassion and true presence (5 items), and moral responsibility (4 items). The Likert 5-point scale was used, with scores ranging from 1 to 5 on a scale of “not at all consistent with me” to “completely consistent with me”, and scores ranging from 21 to 105. Thirteen of the studies further reported mean scores and standard deviations for their four dimensions. (Table 1)

### Nurses’ moral courage

**Fig. 2:**
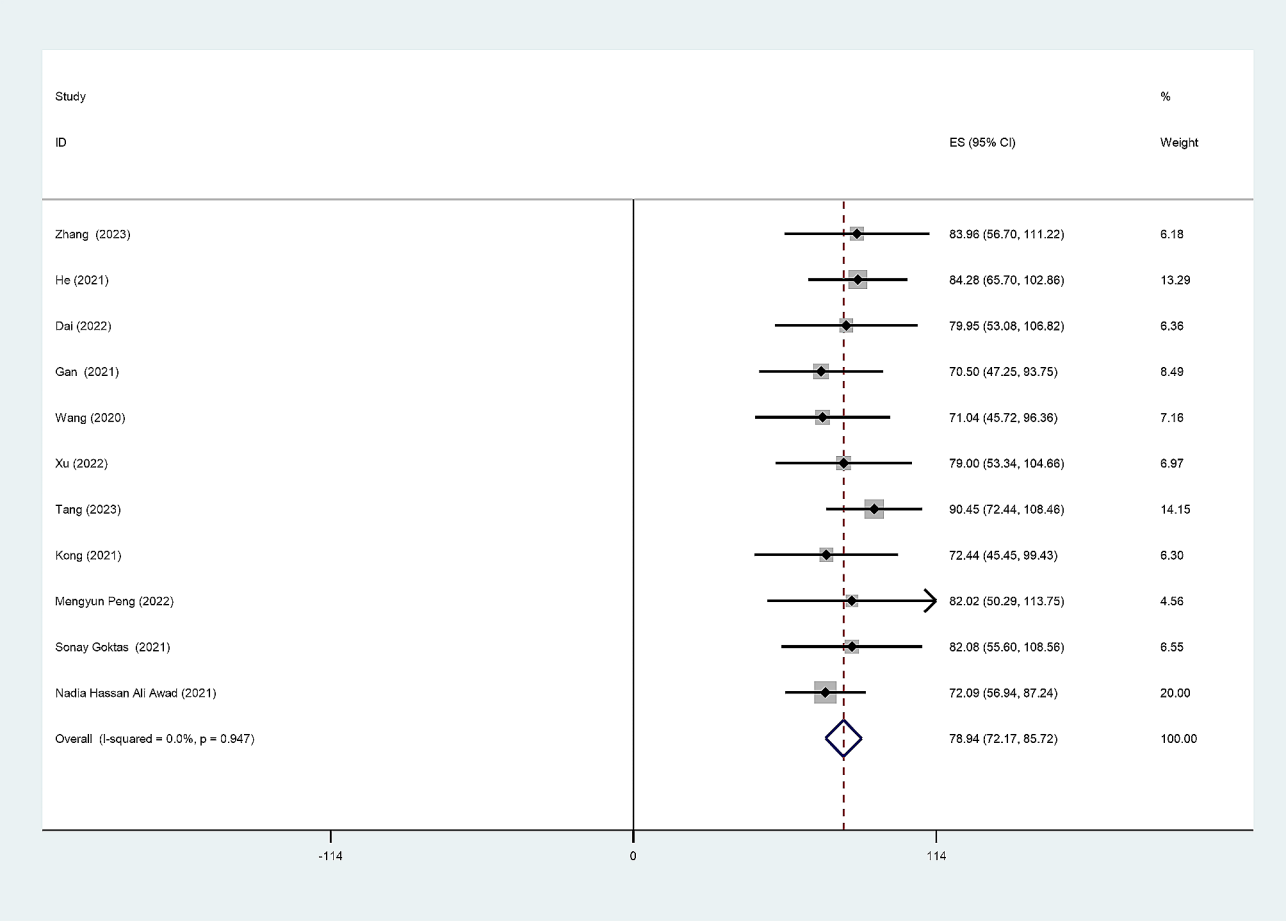
Forest plot of pooled mean scores for total score of moral courage level

**Fig. 3:**
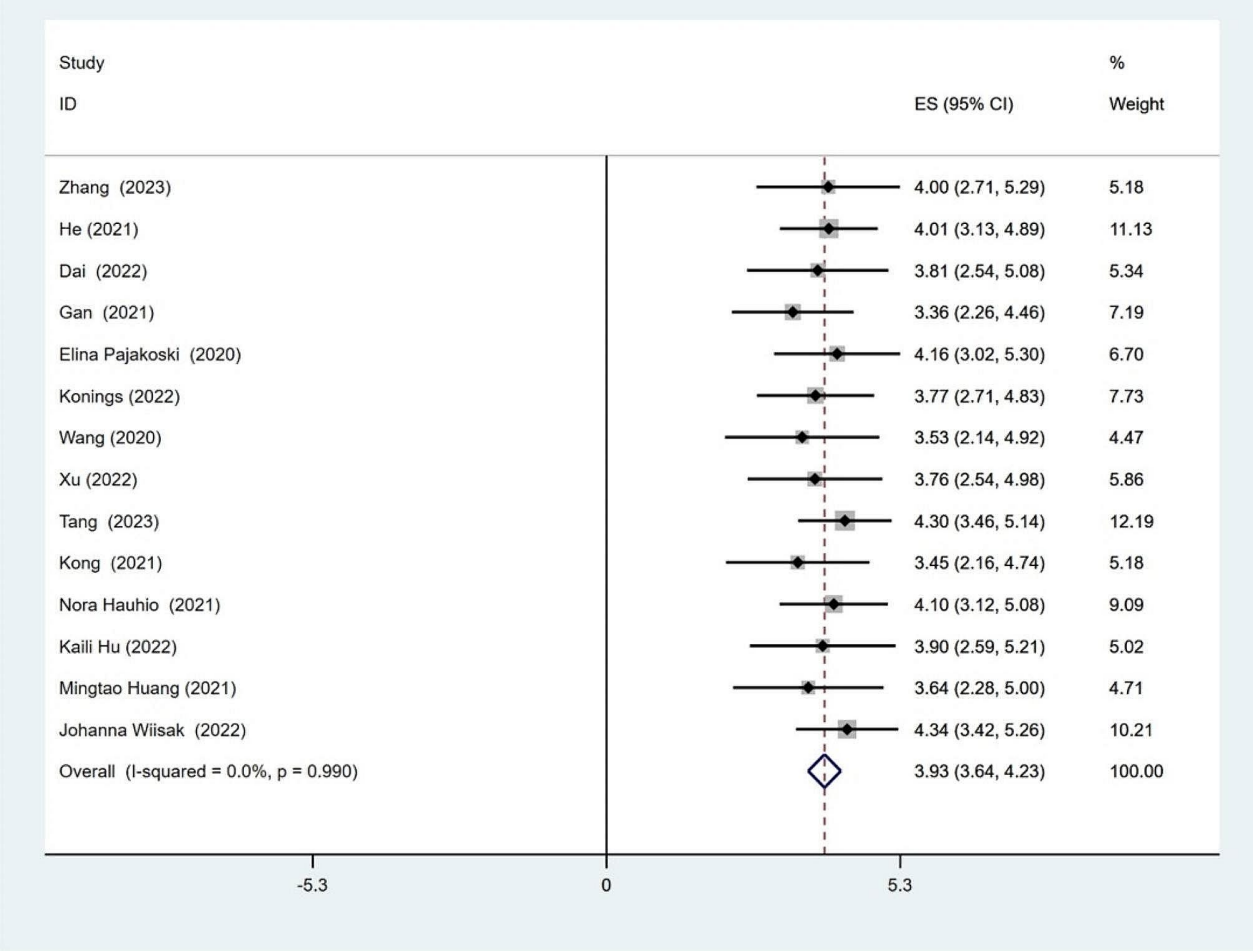
Forest plot of pooled mean scores for average score of entries

Eleven of these studies reported total scores for nurses’ moral courage, Fourteen studies reported mean entry scores for nurses’ moral courage, and the meta-analysis found that the total scores for nurses’ moral courage (*Q* = 4.00, *I*^*2*^ = 0.0%, *p* = 0.947), mean entry scores for nurses’ moral courage (*Q* = 4.07, *I*^*2*^ = 0.0%, *p* = 0.99), and fixed-effects models were used to pool effect sizes. The meta-analysis results showed a pooled mean score were 78.94 (95% CI: 72.17, 85.72), 3.93 (95% CI: 3.64, 4.23). (Figures 2 and 3)

A total of thirteen studies were included for the analysis of the four dimensions. The meta-analysis found that compassion and true presence (*Q* = 4.16, *I*^*2*^ = 0.0%, *p* = 0.998), commitment to good care (*Q* = 4.63, *I*^*2*^ = 0.0%, *p* = 0.969), moral integrity (*Q* = 2.81, *I*^*2*^ = 0.0%, *p* = 0.997), and moral responsibility (*Q* = 2.65, *I*^*2*^ = 0.0%, *p* = 0.998) was homogeneous, and fixed-effects models were used to pool effect sizes, with a pooled mean scores were 3.84 (95% CI: 3.46, 4.21), 3.76 (95% CI: 3.40, 4.12), 3.89 (95% CI: 3.54, 4.24), and 3.84 (95% CI: 3.46, 4.21) respectively. (Supplementary Figs. 1–4)

### Subgroup analyses of moral courage for nurses

The subgroup analysis revealed relatively high level of moral courage among female nurses, nurses with higher education, nurse leaders, nurses who had experienced moral issues or were knowledgeable about them, and nurses who had never considered leaving their jobs. (Table 2)

### Quality appraisal

Eight of the seventeen cross-sectional studies had High methodological quality (AHRQ scores of 8), and nine had moderate methodological quality (AHRQ scores of 6–7). The risk of bias for included studies was mainly from item 2 (The inclusion and exclusion criteria for exposed and unexposed subjects were not listed, or reference was made to previous publications), item 7 (No explanation was given for any patients excluded from the analysis), item 9 (There was no explanation on how to handle missing data in the analysis) and item 11 (The percentage of patients who did not have clear expected follow-up and did not receive incomplete data or follow-up), and all included studies were included in the meta-analysis because they were of moderate to high quality. (Supplementary Table 2)

### Sensitivity analysis/ risk of publication bias

**Fig. 4:**
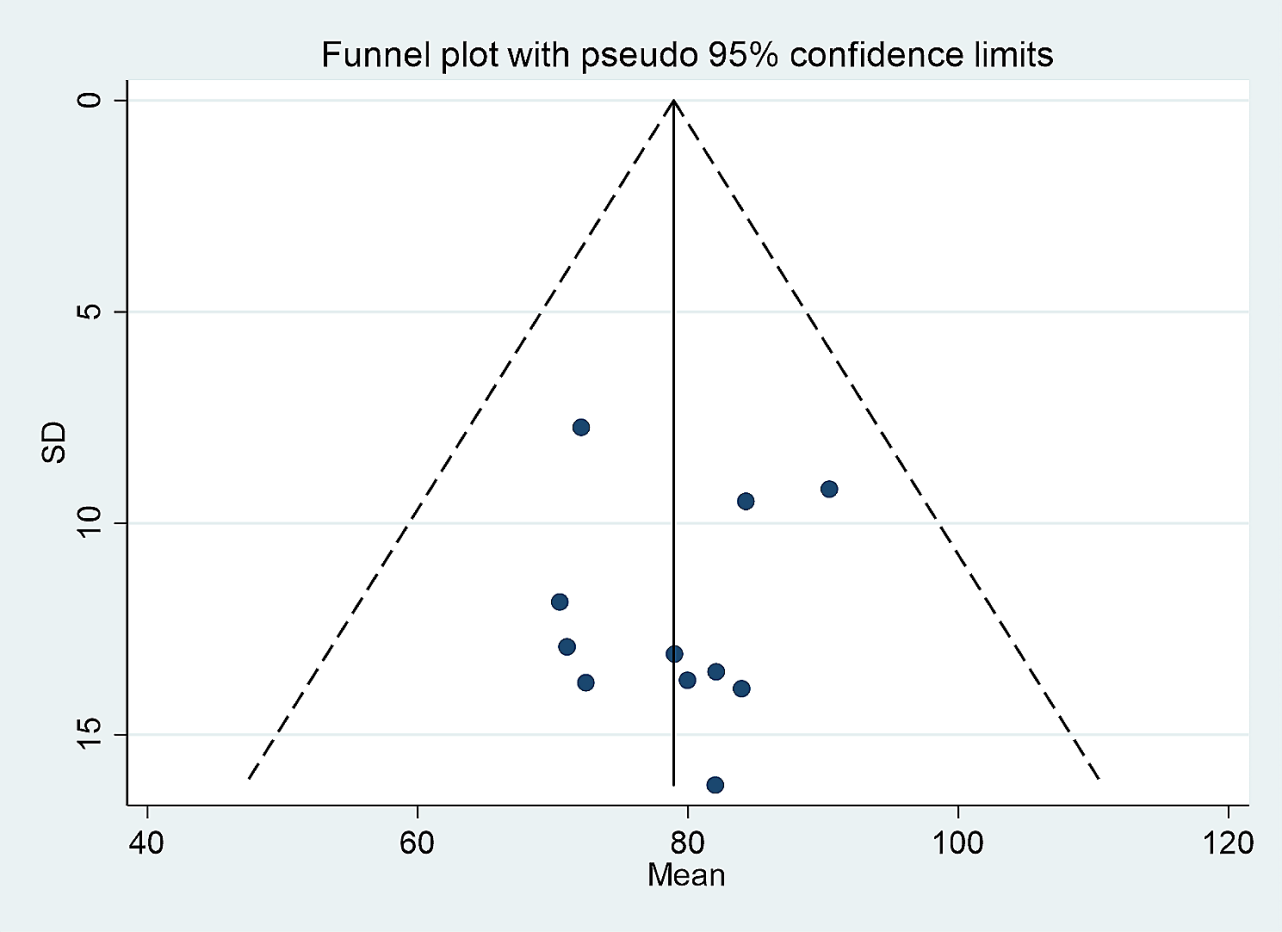
The publication bias in the estimated aggregate average score of moral courage evaluated by the funnel plot

**Fig. 5:**
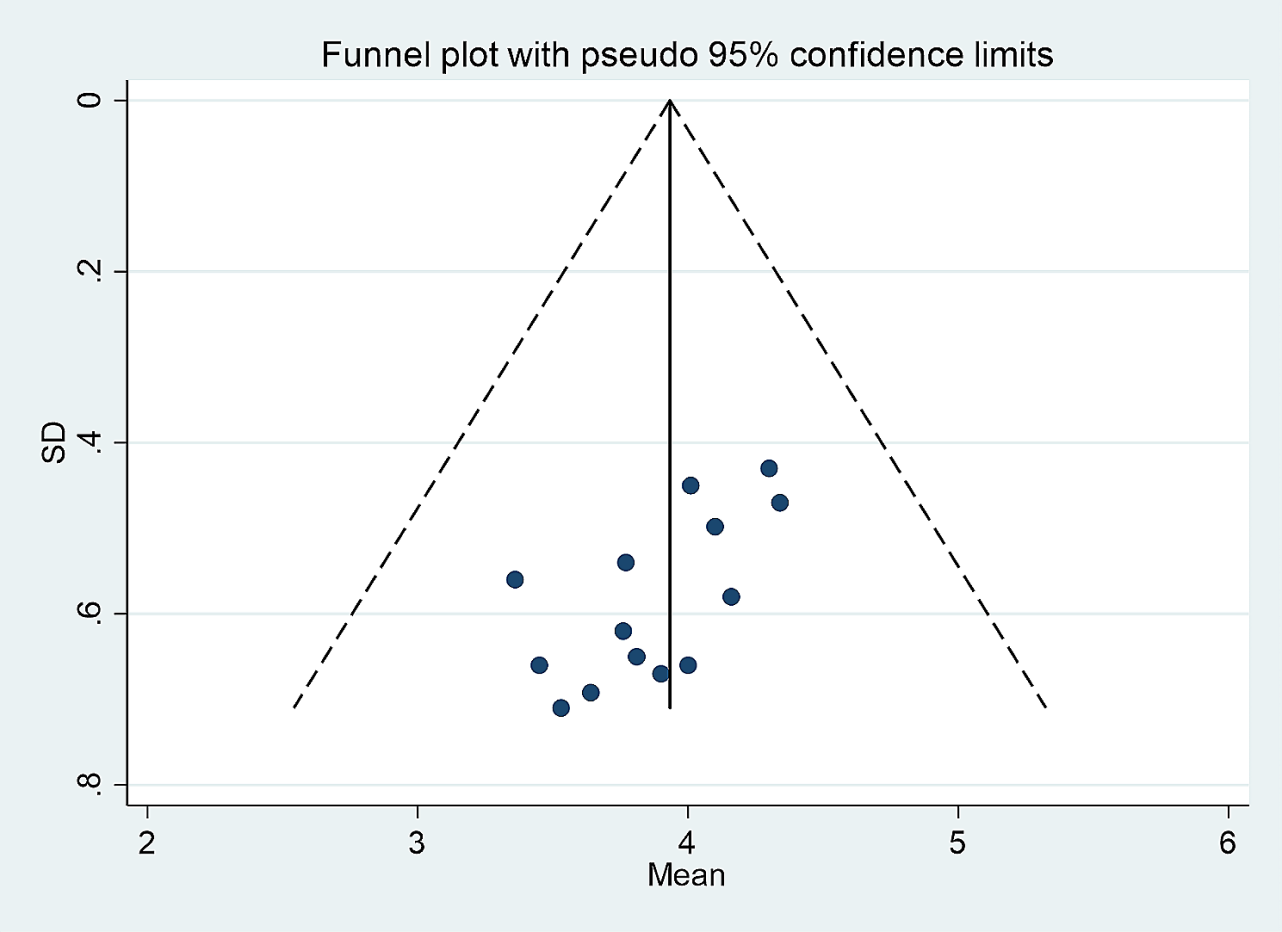
The publication bias of the average score of moral courage items evaluated through a funnel plot

The funnel plot distribution is symmetrical (Figs. 4 and 5), and sensitivity analysis revealed no significant differences between the results and the overall comprehensive estimate, indicating that the meta-analysis findings are relatively stable and reliable (Supplementary Figs. 5–6). Egger’s test result was 0.533 (*p* = 0.993) for the total score of moral courage level for nurses. Therefore, there was no significant publication bias. Egger’s test result was 0.042 (*p* = 0.009) for the mean entry scores for nurses’ moral courage level, in this regard, we performed the cut-and-patch method and the results showed that *P* = 0.99. (Supplementary Figs. 7–9)

## Discussion

The Agency for Healthcare Research and Quality (AHRQ) has recommended quality evaluation criteria for observational studies [25], which assess the risk of bias in 5 domains: selection bias, implementation bias, follow-up bias, measurement bias, and reporting bias. In the cross-sectional studies included in our review, their scores range from 6 to 8, indicating a higher quality of inclusion in the study, the main problem was that the studies lacked exclusion criteria, did not explain the reasons for excluding patients, and did not explain how the analysis handled the missing data. Studies that are rated as high quality are mainly due to their emphasis on sample size and study quality, meaning that they describe any assessments performed to ensure quality and explain the reasons for excluding any patients from the analysis. Therefore, future researchers should pay attention to the above problems when conducting cross-sectional studies.

To our knowledge, this is the first quantitative meta-analysis of nurses’ level of moral courage. In our meta-analysis, we analyzed the four dimensions of the Nurses’ Moral Courage Scale and found that mean scores of all four dimensions were in the moderate to high range. Subgroup analyses to further explore how gender, level of education, ethical experience and related knowledge, and whether resignation was considered affected nurses’ levels of moral courage. By doing so, we aimed to provide a more nuanced understanding of this critical aspect of nursing practice.

This review find that the level of moral courage of nurses is at a medium to high range, the results of this review are similar to the findings of Dai [32] and Xu [36]. This is an encouraging finding as it suggests that many nurses possess the necessary qualities to provide exceptional care for their patients. The four areas of nurses’ moral courage are moral responsibility, compassion and true presence, moral integrity and commitment to good care. These are important components of effective nursing practices, reflecting a deep commitment to the profession. However, there is always room for improvement. Although the current level of moral courage among nurses is commendable, we believe that with the continuous efforts and support of healthcare organizations, this can be further strengthened. By creating an environment that encourages ethical decision-making and prioritizes patient centered care, we can take our nurses to new heights of excellence. In summary, although there is still work to be done to comprehensively improve the moral courage level of nurses, this review provides optimistic reasons for the future of nursing practice.

With continuous attention to these key areas, including moral responsibility, compassion and true presence, moral integrity and commitment to good care, we can continue to build a good medical system.When the dimensions were analyzed and compared, the highest scores were found for moral integrity, Similar to the results of the study by Hu et al. [39].Which focuses on adherence to the basic principles and values of the profession and health care, especially in situations where there is a risk of negative consequences for others [44]. The fact that nurses scored high on this dimension indicates their unwavering commitment to upholding ethical guidelines and demonstrates their courage and ability to act accordingly; commitment to good care is relatively low, similar to the results of Xu et al. [36], Koning et al. [34]. The main content of this dimension refers to nurses’ courage to defend the good rights of patients in the case of insufficient resources or professional competence, compromise or coercive practices that threaten the good care of patients [44]. The low score of this dimension indicates that nurses’ courage to defend the good rights of patients in the case of insufficient resources or professional competence, compromise or coercive practices that threaten the good care of patients Inadequate.

Interestingly, we found that female nurses exhibit higher moral courage than male nurses. This discovery led us to explore the potential reasons for this disparity, assuming that it may be related to the professional identity of male nurses. Our analysis reveals a positive correlation between professional identity and job engagement, indicating that those with stronger professional identity are more likely to participate in their work [45]. However, we also found that male nurses with lower professional identity often exhibit less work enthusiasm, which in turn affects their moral courage. This is an important insight as it emphasizes the need for healthcare organizations to cultivate strong professional identities among all staff, especially male nurses, who may face unique challenges in establishing themselves in a predominantly female field. In a study on the moral courage level of Argentine doctors, we also found that men have lower level of moral courage than women [46]. Subgroup analysis revealed that the higher the level of education, the higher the level of moral courage, which may be related to the fact that nurses with higher education have a higher level of professional knowledge and a better judgment of the treatment and care plan for patients; Meanwhile, our subgroup analysis revealed a large difference in the level of moral courage between nurse leaders and clinical nurses. This may be related to the fact that the professional role of the nurse leader needs to deal with complex nurse-patient and health care relationships on a daily basis, and that he or she has a wider range of interactions at work, has more power, and thus has relatively higher moral courage [32]; Compared to nurses who have experienced moral distress and related knowledge, inexperienced nurses have relatively low levels of moral courage, which may increase with work experience, repeated confrontation with moral challenges, and learning from this may increase with experience, repeated confrontation with ethical challenges, and learning from ethical behavior [47], for example, the relatively high level of moral courage among nurses compared to graduating nursing students may be related to the environment in which the graduating nurses are placed and their age. Clinical nurses often encounter moral dilemmas in their work, which may be associated with their increasing level of moral courage as they gain experience [48]; The higher level of moral courage among nurses who had never considered leaving compared to those who had considered leaving may be related to job dissatisfaction among nurses who considered leaving, this result is similar to the view of Khodaveisi M et al. [15].

Overall, nurses have played a valuable role in promoting ethical practices in the medical environment. They are firmly committed to upholding ethical principles, which not only benefits individual patients but also contributes to building a more just and equitable medical environment. This review suggests that the moral courage level of nurses still needs to be further improved. Therefore, it is imperative that nursing managers and hospital administrators recognize the crucial role of moral courage in the nursing profession. Nurses are often faced with moral distress that require them to make difficult decisions, and having a high level of moral courage can greatly impact their ability to act ethically. To this end, we recommend that senior nurses take an active role in mentoring junior nurses and providing guidance on how to navigate complex ethical situations. By sharing their own experiences and offering support, they can help prevent junior nurses from encountering similar challenges in the future. Additionally, experienced nurses should be encouraged to lead ethics lectures and discussions within their departments. This will not only improve the moral sensitivity of all nurses but also foster a culture of open communication where ethical concerns can be addressed openly and honestly [49]. Ultimately, by prioritizing the development of moral courage among its nursing staff, hospitals can ensure that patients receive care that is both compassionate and ethically sound.

**Table 1:** Basic characteristics of included studies

Study	Country	Study design	Total sample	Gender		Age Mean(SD)	Total score of the scale Mean(SD)	Average score of entries Mean(SD)
				Male	Female			
Zhang et al. (2023)	China	Cross-sectional	422	54	368	31.66 ± 5.69	83.96 ± 13.91	4.00 ± 0.66
He et al. (2021)	China	Cross-sectional	693	17	676	30.84 ± 6.74	84.28 ± 9.48	4.01 ± 0.45
Dai et al. (2022)	China	Cross-sectional	390	13	377	33.89 ± 7.64	79.95 ± 13.71	3.81 ± 0.65
Gan et al. (2021)	China	Cross-sectional	368	37	331	24.01 ± 2.49	70.50 ± 11.86	3.36 ± 0.56
Elina Pajakoski et al. (2020)	Finland	Cross-sectional	205	20	182	42.1	NR	4.16 ± 0.58
Konings et al. (2022)	Belgium	Cross-sectional	559	85	474	NR	NR	3.77 ± 0.54
Wang et al. (2020)	China	Cross-sectional	1094	42	1052	NR	71.04 ± 12.92	3.53 ± 0.71
Xu et al. (2022)	China	Cross-sectional	305	102	203	35.27 ± 6.50	79.00 ± 13.09	3.76 ± 0.62
Tang et al. (2023)	China	Cross-sectional	331	77	254	33.34 ± 7.52	90.45 ± 9.19	4.30 ± 0.43
Kong et al. (2021)	China	Cross-sectional	232	3	229	NR	72.44 ± 13.77	3.45 ± 0.66
Nora Hauhio et al. (2021)	Finland	Cross-sectional	482^a^	51	427	41.00 ± 10.7	NR	4.1 ± 0.498
Mengyun Peng et al. (2022)	China	Cross-sectional	781	7	774	33.22 ± 7.22	82.02 ± 16.19	NR
Kaili Hu et al. (2022)	China	Cross-sectional	226	0	226	34.54 ± 5.68	NR	3.9 ± 0.67
Sonay Goktas et al. (2021)	Turkey	Cross-sectional	362	66	296	21.60 ± 4.24	82.08 ± 13.51	NR
Nadia Hassan Ali Awad et al. (2021)	Egypt	Cross-sectional	235	15	220	34.20 ± 7.72	72.09 ± 7.73	NR
Mingtao Huang et al. (2021)	China	Cross-sectional	583	NR	NR	NR	NR	3.64 ± 0.692
Johanna Wiisak et al. (2022)	Finland	Cross-sectional	454	21	428	47.00 ± 11.2	NR	4.34 ± 0.47

Note: SD, standard deviation; NR, not reported.

^a^4 people were missing in the gender survey

**Table 2:** Subgroup analysis of the level of moral courage for nurses

Subgroups	Number of studies	Sample size	Effect mode	Pooled mean score		Effect size		Heterogeneity		Test for subgroup difference
				(95%CI)		Z (p)		*I* ^2^ *(p)*		Z df (p)
Gender										
Male	4	213	Fixed	73.03 (60.66,85.39)		11.58 (< 0.001)		0.0% (0.947)		Z = 16.31 df = 7 (*p* < 0.001)
Female	4	1780	Fixed	76.74 (63.65,89.82)		11.49 (< 0.001)		0.0% (0.903)		
Administrators										
No	6	2473	Fixed	80.19 (70.25,90.12)		15.82 (< 0.001)		0.0% (0.820)		Z = 31.18 df = 11 (*p* < 0.001)
Yes	6	182	Fixed	90.23 (83.66,96.80)		26.92 (< 0.001)		0.0% (0.855)		
Education level										
High school	4	325	Fixed	79.00 (67.24,90.75)		13.17 (< 0.001)		0.0% (0.543)		Z = 19.29 df = 7 (*p* < 0.001)
Bachelor’s degree	4	1618	Fixed	82.02 (70.61,93.43)		14.09 (< 0.001)		0.0% (0.504)		
Experiencing ethical problems or related knowledge										
No	3	579	Fixed	74.60 (58.70,90.50)		9.20 (< 0.001)		0.0% (0.667)		Z = 13.07 df = 4 (*p* < 0.001)
Yes	3	1299	Fixed	79.89 (65.61,94.16)		10.97 (< 0.001)		0.0% (0.765)		
Thinking about resigning										
No	2	434	Fixed	82.42 (63.58,101.26)		8.57 (< 0.001)		0.0% (0.987)		Z = 12.09 df = 3 (*p* < 0.001)
Yes	2	315	Fixed	79.52 (61.23,97.78)		8.53 (< 0.001)		0.0% (0.851)		

### Limitations

There are certain limitations to this review. First, the included studies were cross-sectional in design, therefore, no causal relationship can be inferred from the observed association and inevitably had design flaws. Second, the scales we included were patient self-reported outcome scales, which are somewhat subjective. Third, we did not search the gray literature base and may have missed those unpublished papers. Fourth, in the meta-analysis, scales not developed by Numminen et al. were excluded, which may bias the integration results. Finally, more of the included studies were conducted in China (*n* = 11), thus, the scope of our study may have been limited.

### Clinical implication

This meta-analysis delves into the key topic of nurses’ moral courage. By incorporating relevant literature, this review reveals the current status of nurses’ moral courage level and provides valuable insights for nursing managers and hospital managers. The findings of this meta-analysis have profound implications for healthcare organizations. By better understanding the factors that contribute to moral courage, hospitals can develop effective management strategies to improve ethical practices and strengthen patient care. A key suggestion is to create a positive work environment that supports professional ethics. When nurses feel supported by colleagues and superiors, they are more likely to demonstrate moral courage in challenging situations. Conversely, it can also provide better care for patients and improve the overall quality of care [50]. In addition to creating a supportive workplace culture, hospitals should also prioritize providing relevant training and education around ethical issues. Overall, this meta-analysis represents an important step forward in understanding the moral courage of nurses. By taking action based on these findings, hospitals can create a more ethical workplace culture that benefits both patients and nurses.

## Conclusion

This review find that the level of moral courage of nurses is at a medium to high range, the level of moral courage was lower among nurses who were male, non-nursing managers, had lower education, had not experienced ethical issues, and were considering resignation. These subgroup analysis results indicate that there is still room for improvement in cultivating an environment where all nurses have the right to act on behalf of the best interests of patients. So it is recommended that nursing managers as well as hospital administrators take appropriate measures to create a good working environment for nurses and improve their level of moral courage in order to improve the quality of care.

### Electronic supplementary material

Below is the link to the electronic supplementary material.

Supplementary Material 1

## Data Availability

All the data are available from the corresponding author up on a reasonable request.

## References

[1] Haahr A, Norlyk A, Martinsen B, Dreyer P. Nurses experiences of ethical dilemmas: a review. Nurs Ethics. 2020;27(1):258–72. 30975034 10.1177/0969733019832941

[2] Jena LK, Sarkar J, Goyal S. Sense of courage: the mediating role of courage between emotional reflexivity and work-life integration among nurses in Indian hospitals. Int J Nurs Sci. 2021;8(3):318–24. 34307781 10.1016/j.ijnss.2021.06.001 PMC8283716

[3] Grosek Š, Kučan R, Grošelj J, Oražem M, Grošelj U, Erčulj V, Lajovic J, Borovečki A, Ivanc B. The first nationwide study on facing and solving ethical dilemmas among healthcare professionals in Slovenia. PLoS ONE. 2020;15(7):e0235509. 32663206 10.1371/journal.pone.0235509 PMC7360038

[4] Hally SM, Settle M, Nelson BD. Relationship between Moral distress and intent to leave a position among neonatal intensive care nurses. Adv Neonatal Care. 2021;21(6):E191–8. 34054013 10.1097/ANC.0000000000000891

[5] Huang L, Lei W, Xu F, Liu H, Yu L. Emotional responses and coping strategies in nurses and nursing students during Covid-19 outbreak: a comparative study. PLoS ONE. 2020;15(8):e0237303. 32764825 10.1371/journal.pone.0237303 PMC7413410

[6] Lake ET, Narva AM, Holland S, et al. Hospital nurses’ moral distress and mental health during COVID-19. J Adv Nurs. 2022;78(3):799–809. 34402538 10.1111/jan.15013 PMC8447301

[7] Ulrich C, O’Donnell P, Taylor C, Farrar A, Danis M, Grady C. Ethical climate, ethics stress, and the job satisfaction of nurses and social workers in the United States. Soc Sci Med. 2007;65(8):1708–19. 17619068 10.1016/j.socscimed.2007.05.050 PMC2442035

[8] Maiden J, Georges JM, Connelly CD. Moral distress, compassion fatigue, and perceptions about medication errors in certified critical care nurses. Dimens Crit Care Nurs. 2011 Nov-Dec;30(6):339–45. 10.1097/DCC.0b013e31822fab2a 21983510

[9] Mason VM, Leslie G, Clark K, Lyons P, Walke E, Butler C, Griffin M. Compassion fatigue, moral distress, and work engagement in surgical intensive care unit trauma nurses: a pilot study. Dimens Crit Care Nurs. 2014 Jul-Aug;33(4):215–25. 10.1097/DCC.0000000000000056 24895952

[10] Malliarou M, Nikolentzos A, Papadopoulos D, Bekiari T, Sarafis P. ICU nurse’s Moral Distress as an Occupational Hazard threatening Professional Quality of Life in the time of Pandemic COVID 19. Mater Sociomed. 2021;33(2):88–93. 34483734 10.5455/msm.2021.33.88-93 PMC8385730

[11] Namadi F, Shahbaz A, Jasemi M. Nurses’ lived experiences of Moral courage inhibitors: a qualitative descriptive study. SAGE Open Nurs. 2023;9:23779608231157326. 36844423 10.1177/23779608231157326 PMC9944332

[12] Jiang Fubin, Wang Zhen. A review of research on the formation and effects of moral courage in the workplace. J Manage 2023,20(01):149–58.

[13] Numminen O, Repo H, Leino-Kilpi H. Moral courage in nursing: a concept analysis. Nurs Ethics. 2017;24(8):878–91. 27005953 10.1177/0969733016634155

[14] Safarpour H, Ghazanfarabadi M, Varasteh S, Bazyar J, Fuladvandi M, Malekyan L. The Association between Moral Distress and Moral Courage in nurses: a cross-sectional study in Iran. Iran J Nurs Midwifery Res. 2020;25(6):533–8. 33747844 10.4103/ijnmr.IJNMR_156_19 PMC7968592

[15] Khodaveisi M, Oshvandi K, Bashirian S, Khazaei S, Gillespie M, Masoumi SZ, Mohammadi F. Moral courage, moral sensitivity and safe nursing care in nurses caring of patients with COVID-19. Nurs Open. 2021;8(6):3538–46. 33945661 10.1002/nop2.903 PMC8242869

[16] Tang YC, Wang CS, Zuo ZM, Lv LP, Zhu WZ, Tian YP. Analysis of the current situation and influencing factors of moral courage among psychiatric nurses[J]. Gen Pract Nurs. 2023;21(06):848–52.

[17] Gan L, Li H, Liu XQ. A study on the current situation and influencing factors of professional deviant behaviors of junior nurses[J]. J Nurs 2021,36(22):12–5.

[18] Hauhio N, Leino-Kilpi H, Katajisto J, Numminen O. Nurses’ self-assessed moral courage and related socio-demographic factors. Nurs Ethics 2021 Nov-Dec;28(7–8):1402–15. 10.1177/0969733021999763 34100317

[19] Escolar-Chua RL. Moral sensitivity, moral distress, and moral courage among baccalaureate Filipino nursing students. Nurs Ethics. 2018;25(4):458–69. 27364536 10.1177/0969733016654317

[20] Pajakoski E, Rannikko S, Leino-Kilpi H, Numminen O. Moral courage in nursing - an integrative literature review. Nurs Health Sci. 2021;23(3):570–85. 33389792 10.1111/nhs.12805

[21] Santos RPD, Garros D, Carnevale F. Difficult decisions in pediatric practice and moral distress in the intensive care unit. Rev Bras Ter Intensiva 2018 Apr-Jun;30(2):226–32. 10.5935/0103-507X.20180039 PMC6031410 29995089

[22] Page MJ, McKenzie JE, Bossuyt PM, Boutron I, Hoffmann TC, Mulrow CD et al. The PRISMA 2020 statement: an updated guideline for reporting systematic reviews BMJ. 2021; 372:n71. 10.1136/bmj.n71 PMC8005924 33782057

[23] Stroup DF, Berlin JA, Morton SC, Olkin I, Williamson GD, Rennie D, Moher D, Becker BJ, Sipe TA, Thacker SB. Meta-analysis of observational studies in epidemiology: a proposal for reporting. Meta-analysis of Observational studies in Epidemiology (MOOSE) group. JAMA. 2000;283(15):2008–12. 10789670 10.1001/jama.283.15.2008

[24] Numminen O, Katajisto J, Leino-Kilpi H. Development and validation of Nurses’ Moral Courage Scale. Nurs Ethics 2019 Nov-Dec;26(7–8):2438–55. 10.1177/0969733018791325 30185132

[25] Rostom A, Dubé C, Cranney A, Saloojee N, Sy R, Garritty C, Sampson M, Zhang L, Yazdi F, Mamaladze V, Pan I, McNeil J, Moher D, Mack D, Patel D. Celiac disease. Evid Rep Technol Assess (Summ). 2004;(104):1–6. PMC4781297 15346868

[26] Zeng X, Zhang Y, Kwong JS, Zhang C, Li S, Sun F, Niu Y, Du L. The methodological quality assessment tools for preclinical and clinical studies, systematic review and meta-analysis, and clinical practice guideline: a systematic review. J Evid Based Med. 2015;8(1):2–10. 25594108 10.1111/jebm.12141

[27] Rostom A, Dubé C, Cranney A et al. Celiac Disease. Rockville (MD): Agency for Healthcare Research and Quality (US); 2004 Sep. (evidence Reports/Technology assessments, No. 104.) Appendix D. Quality Assessment Forms.

[28] Higgins JP, Thompson SG, Deeks JJ, Altman DG. Measuring inconsistency in meta-analyses. BMJ. 2003;327(7414):557–60. 12958120 10.1136/bmj.327.7414.557 PMC192859

[29] Egger M, Davey Smith G, Schneider M, Minder C. Bias in meta-analysis detected by a simple, graphical test. BMJ. 1997;315(7109):629–34. 9310563 10.1136/bmj.315.7109.629 PMC2127453

[30] Zhang YD, Du AP, Tian Y. Analysis of the current situation and influencing factors of moral courage of ICU nurses[J]. J Nurs 2023,38(02):51–4.

[31] He J, Yang X, Xia HY, Xiang XY, Han WL, Wang DH. Analysis of the current situation and influencing factors of moral courage among 693 clinical nurses in a secondary hospital[J]. J Nurs 2021,28(18):41–5.

[32] Dai HM, Li Y, Wu HY. Study on the correlation between moral courage and moral dilemma of nursing staff [J]. J Nurs. 2022;37(21):53–7.

[33] Pajakoski E, Rannikko S, Leino-Kilpi H, Eliisa, Löyttyniemi. Olivia Numminen. Nurses’ moral courage in Finnish older people care: a cross-sectional study[J]. Nordic J Nurs Res. 2023;43(1):20571585231162807.

[34] Konings KJ, Gastmans C, Numminen OH, Claerhout R, Aerts G, Leino-Kilpi H, de Casterlé. BD. Measuring nurses’ moral courage: an explorative study. Nurs Ethics. 2022;29(1):114–130. 10.1177/09697330211003211 34278853

[35] Wang SY, Jiang WB, Liu C, Shang QW, Yang HP, Zhang Y, Wei LL. A study on the moral courage of nurses and its influencing factors [J]. Chin J Nurs Educ. 2022;19(08):732–6.

[36] Xu J, Han L, Wang J, Pao YJ, Deng JQ, Huang ZW. Analysis of moral courage and influencing factors of psychiatric nurses [J]. J Nurs. 2022;37(16):51–3.

[37] Linshu K, Jingyan W, Lina D, et al. Investigation on the Moral courage of clinical nurses in geriatric hospitals [J]. J Hosp Manage People’s Liberation Army. 2021;28(04):383–5.

[38] Peng M, Saito S, Guan H, Li B. Moral distress, moral courage, and career identity among nurses: a cross-sectional study. Nurs Ethics 2022 Dec 22:9697330221140512. 10.1177/09697330221140512 36545793

[39] Hu K, Liu J, Zhu L, Zhou Y. Clinical nurses’ moral courage and related factors: an empowerment perspective. BMC Nurs. 2022;21(1):321. 36419129 10.1186/s12912-022-01093-9 PMC9685865

[40] Goktas S, Aktug C, Gezginci E. Evaluation of moral sensitivity and moral courage in intensive care nurses in Turkey during the COVID-19 pandemic. Nurs Crit Care. 2023;28(2):261–71. 35821613 10.1111/nicc.12820 PMC9350110

[41] Ali Awad NH, Al-Anwer Ashour HM. Crisis, ethical leadership and moral courage: ethical climate during COVID-19. Nurs Ethics. 2022;29(6):1441–56. 35724327 10.1177/09697330221105636 PMC9209857

[42] Huang M, Dong W, Zhao Q, Mo N. Factors associated with the moral courage of nurses in China: a cross-sectional study. Nurs Open. 2023 Feb 22. 10.1002/nop2.1672 PMC10277425 36811339

[43] Wiisak J, Suhonen R, Leino-Kilpi H. Whistle-blowers - morally courageous actors in health care? Nurs Ethics. 2022;29(6):1415–29. 35727204 10.1177/09697330221092341 PMC9527363

[44] Wang SY, Wei LL, Zhang Y, Li T, Jiang WB, Yang HP, Chen K, Wang SY, Chen QQ. Sinicization and reliability testing of the Nurse Moral courage Scale[J]. J Nurs 2019,34(21):92–5.

[45] Wu C, Fu MM, Cheng SZ, Lin YW, Yan JR, Wu J, Zhang XY, Cao BH, Du J, Lang HJ. Career identity and career success among Chinese male nurses: the mediating role of work engagement. J Nurs Manag. 2022;30(7):3350–9. 36056581 10.1111/jonm.13782 PMC10087454

[46] Borracci RA, Ciambrone G, Gallesio JMA. Correlation between moral courage score and social desirability score of the medical residents and fellows in Argentina. J Educ Eval Health Prof. 2020;17:6. 32079053 10.3352/jeehp.2020.17.6 PMC7364024

[47] Ren P, Yin F, Jiang XR, Luo YY, An D. Research progress of moral courage of nurses[J]. Gen Pract Nurs 2021,19(35):4951–4.

[48] Koskinen S, Pajakoski E, Fuster P, Ingadottir B, Löyttyniemi E, Numminen O, Salminen L, Scott PA, Stubner J, Truš M, Leino-Kilpi H, ProCompNurse Consortium. Analysis of graduating nursing students’ moral courage in six European countries. Nurs Ethics. 2021;28(4):481–97. 33118442 10.1177/0969733020956374 PMC8182296

[49] Ko HK, Tseng HC, Chin CC, Hsu MT. Phronesis of nurses: a response to moral distress. Nurs Ethics. 2020;27(1):67–76. 30975049 10.1177/0969733019833126

[50] Fradelos C, Alexandropoulou E, Kontopoulou CA, Alikari L, Papagiannis V, Tsaras D, Papathanasiou K. The effect of hospital ethical climate on nurses’ work-related quality of life: a cross-sectional study. Nurs Forum. 2022;57(2):244–51. 34773637 10.1111/nuf.12671

