# Supplementary material for: Moral courage level of nurses: a systematic review and meta-analysis
Source: BMC Nurs. 2024 Aug 2;23:530. doi: 10.1186/s12912-024-02082-w (PMC11295526; doi:10.1186/s12912-024-02082-w)
Supplement: Supplementary file 1 — Supplementary Material 1 [file 12912_2024_2082_MOESM1_ESM.docx]

**Supplementary table 1**

**Systematic literature review search strategy in PubMed, Web of science, EMBASE, CINAHL, Cochrane Library**

| **Database** | **Index and keyword terms** |
| --- | --- |
| PubMed | #1TS=(Nurses OR Nurse OR Personnel, Nursing OR Nursing Personnel OR Registered Nurses OR Nurse, Registered OR Nurses, Registered OR Registered Nurse)  #2TS=(Moral Courage)  #3 #1 AND #2 |
| Web of science | #1TS=(Nurses OR Nurse OR Personnel, Nursing OR Nursing Personnel OR Registered Nurses OR Nurse, Registered OR Nurses, Registered OR Registered Nurse)  #2TS=(Moral Courage)  #3 #1 AND #2 |
| EMBASE | #4 'moral courage':ab,ti  #3 #1 OR #2  #2 'nurse*':ab,ti OR 'personnel, nursing':ab,ti OR 'nursing personnel':ab,ti OR 'registered nurses':ab,ti OR 'nurse, registered':ab,ti OR 'nurses, registered':ab,ti OR 'registered nurse':ab,ti  #1 'nurse'/exp |
| CINAHL | S1 TI nursing personnel OR TI registered nurses OR TI nurse, registered OR TI nurses, registered OR TI registered nurse OR TI personnel, nursing OR TI nurse*  S2 TI Moral Courage |
| Cochrane Library | #1 MeSH descriptor: [Nursing] in all MeSH products  #2 (nurse*):ti,ab,kw OR (personnel, nursing):ti,ab,kw OR (nursing personnel):ti,ab,kw OR (registered nurses):ti,ab,kw OR (nurse, registered):ti,ab,kw  #3 (nurses, registered):ti,ab,kw OR (registered nurse):ti,ab,kw  #4 (moral courage):ti,ab,kw  #5 #1 OR #2 OR #3  #6 #5 AND #4 |

**Supplementary table 2**

**Quality assessment of included studies**

| Stady | Item 1 | Item 2 | Item 3 | Item 4 | Item 5 | Item 6 | Item 7 | Item 8 | Item 9 | Item 10 | Item 11 | Total score | Quality |
| --- | --- | --- | --- | --- | --- | --- | --- | --- | --- | --- | --- | --- | --- |
| Zhang et al (2023) | Y | N | Y | Y | Y | Y | N | Y | N | Y | U | 7 | M |
| He et al (2021) | Y | N | Y | Y | Y | Y | Y | Y | N | Y | U | 8 | H |
| Dai et al (2022) | Y | N | Y | Y | Y | Y | Y | Y | N | Y | U | 8 | H |
| Gan et al (2021) | Y | N | Y | Y | Y | Y | Y | Y | N | Y | U | 8 | H |
| Elina Pajakoski et al (2020) | Y | N | Y | Y | Y | N | N | Y | N | Y | U | 6 | M |
| Konings et al (2022) | Y | N | Y | Y | Y | N | N | Y | N | Y | U | 6 | M |
| Wang et al (2020) | Y | N | Y | Y | Y | Y | Y | Y | N | Y | U | 8 | H |
| Xu et al (2022) | Y | N | Y | Y | Y | Y | Y | Y | N | Y | U | 8 | H |
| Tang et al (2023) | Y | N | Y | Y | Y | N | Y | Y | N | Y | U | 7 | H |
| Kong et al (2021) | Y | N | Y | Y | Y | Y | Y | Y | N | Y | U | 8 | H |
| Nora Hauhio et al (2021) | Y | N | Y | Y | Y | Y | N | Y | N | Y | U | 7 | M |
| Mengyun Peng et al (2022) | Y | N | Y | Y | Y | Y | Y | Y | N | Y | U | 8 | H |
| Kaili Hu et al (2022) | Y | N | Y | Y | Y | Y | Y | Y | N | Y | U | 8 | H |
| Sonay Goktas et al (2021) | Y | N | Y | Y | Y | Y | N | Y | N | Y | U | 7 | M |
| Nadia Hassan Ali Awad et al (2021) | Y | N | Y | Y | Y | N | N | Y | N | Y | U | 6 | M |
| Mingtao Huang et al (2021) | Y | N | Y | Y | Y | Y | N | Y | N | Y | U | 7 | M |
| Johanna Wiisak et al (2022) | Y | N | Y | Y | Y | N | N | Y | N | Y | U | 6 | M |
| Note: Y, yes; N, no; U, unclear; H, high quality; M, medium quality. | | | | | | | | | | | | | |
| Item 1: Define the source of information (survey, record review). | | | | | | | | | | | | | |
| Item 2: List inclusion and exclusion criteria for exposed and unexposed subjects (cases and controls) or refer to previous publications. | | | | | | | | | | | | | |
| Item 3: Indicate time period used for identifying patients. | | | | | | | | | | | | | |
| Item 4: Indicate whether or not subjects were consecutive if not population-based. | | | | | | | | | | | | | |
| Item 5: Indicate if evaluators of subjective components of study were masked to other aspects of the status of the participants. | | | | | | | | | | | | | |
| Item 6: Describe any assessments undertaken for quality assurance purposes (e.g., test/retest of primary outcome measurements). | | | | | | | | | | | | | |
| Item 7: Explain any patient exclusions from analysis. | | | | | | | | | | | | | |
| Item 8: Describe how confounding was assessed and/or controlled. | | | | | | | | | | | | | |
| Item 9: If applicable, explain how missing data were handled in the analysis. | | | | | | | | | | | | | |
| Item 10: Summarize patient response rates and completeness of data collection. | | | | | | | | | | | | | |
| Item 11: Clarify what follow-up, if any, was expected and the percentage of patients for which incomplete data or follow-up was obtained. | | | | | | | | | | | | | |


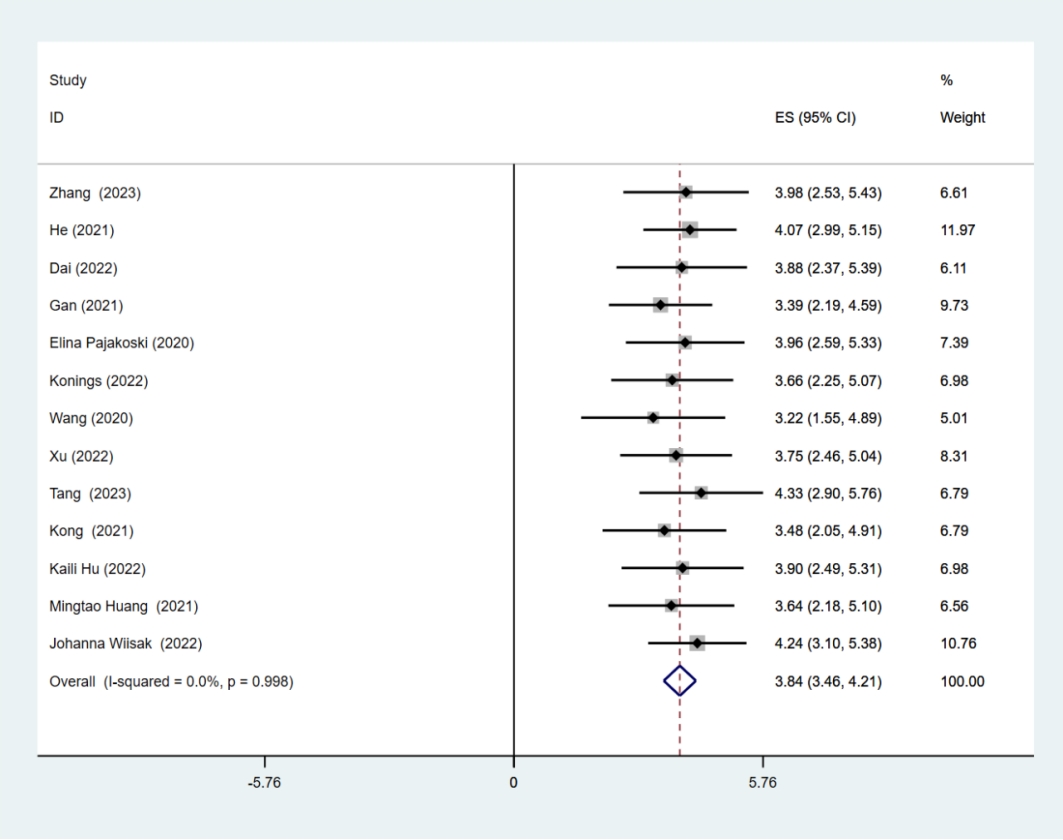


**Supplementary figure 1. Forest plot of pooled mean scores for compassion and true presence.**


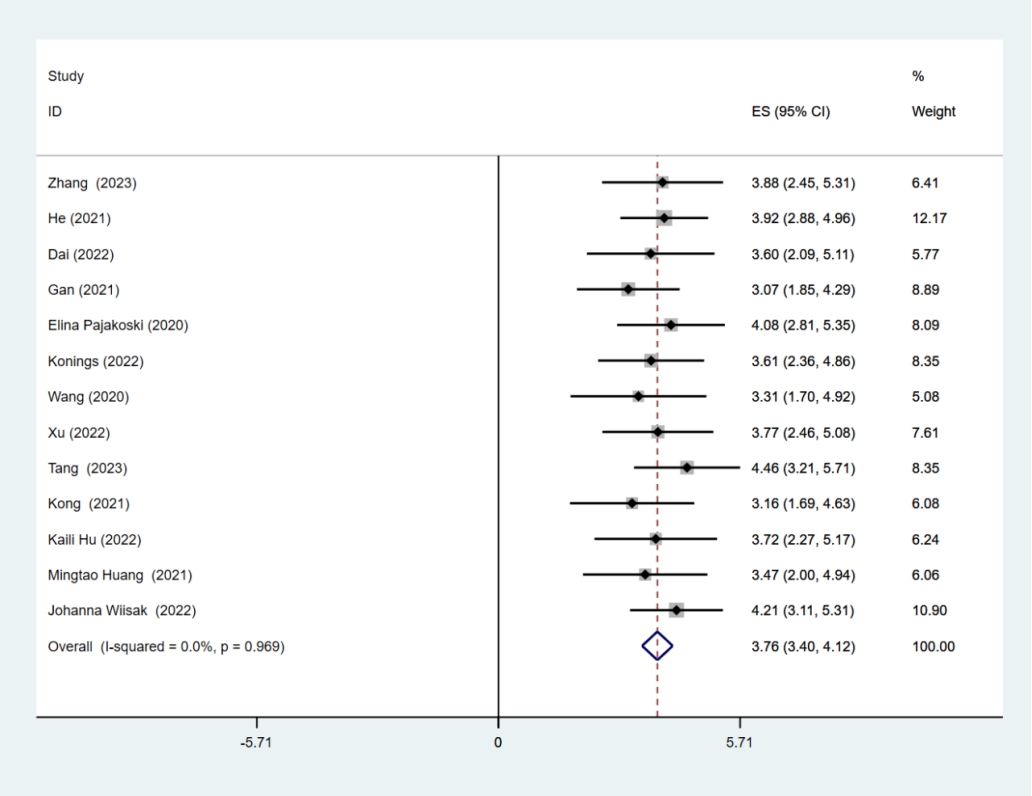


**Supplementary figure 2. Forest plot of pooled mean scores for commitment to good care.**


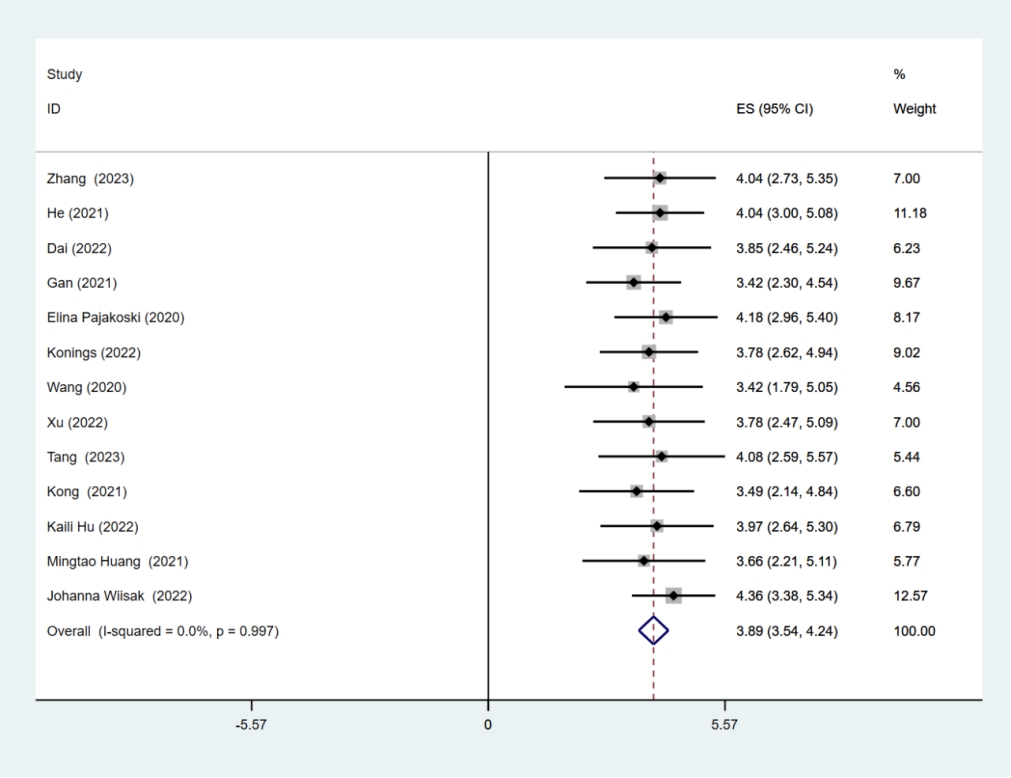


**Supplementary figure 3. Forest plot of pooled mean scores for moral integrity.**


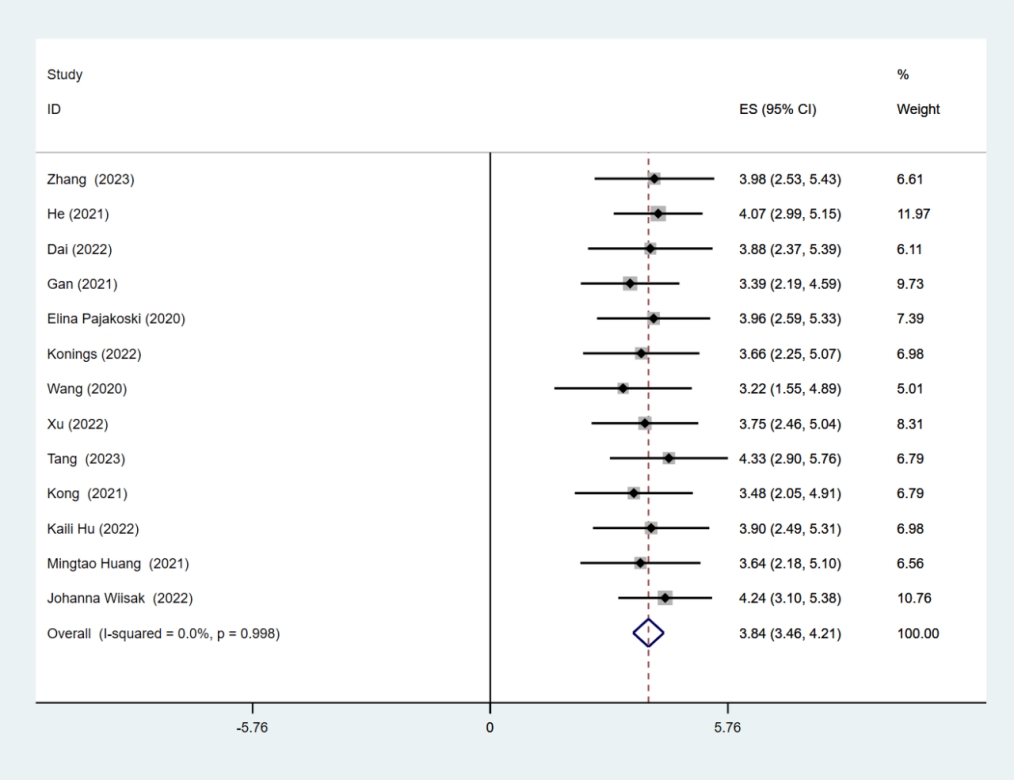


**Supplementary figure 4. Forest plot of pooled mean scores for moral responsibility.**

**Supplementary figure 5. Sensitivity analysis of the total score of nurses' moral courage.**

**Supplementary figure 6. Sensitivity analysis of the average score of each item on nurses' moral courage.**

**Supplementary figure 7. The Publication bias of the total mean score of moral courage level.**

**Supplementary figure 8. The Publication bias of the mean entry scores for nurses' moral courage level.**


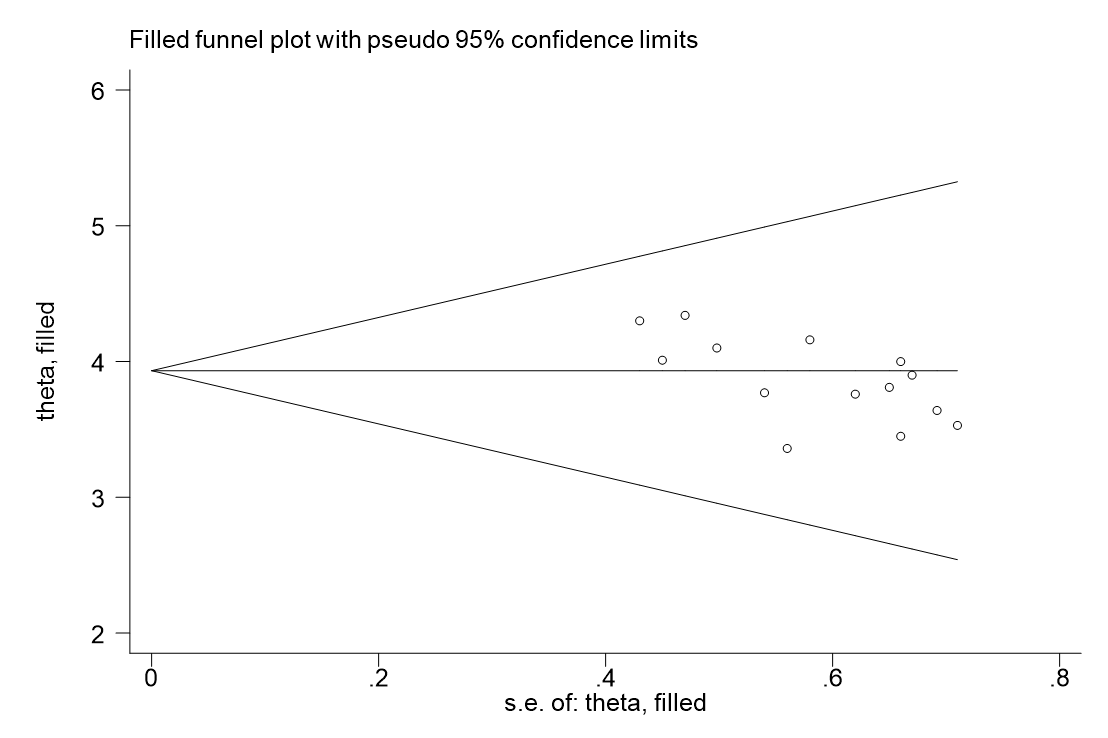


**Supplementary figure 9. Cut and replace the hair correction nurse moral courage level each entry mean score.**
